# Adoption of artificial intelligence and machine learning in banking systems: a qualitative survey of board of directors

**DOI:** 10.3389/frai.2024.1440051

**Published:** 2024-11-27

**Authors:** Abdullah Eskandarany

**Affiliations:** College of Business, University of Jeddah, Jeddah, Saudi Arabia

**Keywords:** artificial intelligence, machine learning, stakeholder theory, board of directors, banking sector, Saudi Arabia

## Abstract

The aim of the paper is twofold. First to examine the role of the board of directors in facilitating the adoption of AI and ML in Saudi Arabian banking sector. Second, to explore the effectiveness of artificial intelligence and machine learning in protection of Saudi Arabian banking sector from cyberattacks. A qualitative research approach was applied using in-depth interviews with 17 board of directors from prominent Saudi Arabian banks. The present study highlights both the opportunities and challenges of integrating artificial intelligence and machine learning advanced technologies in this highly regulated industry. Findings reveal that advanced artificial intelligence and machine learning technologies offer substantial benefits, particularly in areas like threat detection, fraud prevention, and process automation, enabling banks to meet regulatory standards and mitigate cyber threats efficiently. However, the research also identifies significant barriers, including limited technological infrastructure, a lack of cohesive artificial intelligence strategies, and ethical concerns around data privacy and algorithmic bias. Interviewees emphasized the board of directors’ critical role in providing strategic direction, securing resources, and fostering partnerships with artificial intelligence technology providers. The study further highlights the importance of aligning artificial intelligence and machine learning initiatives with national development goals, such as Saudi Vision 2030, to ensure sustained growth and competitiveness. The findings from the present study offer valuable implications for policymakers in banking in navigating the complexities of artificial intelligence and machine learning adoption in financial services, particularly in emerging markets.

## 1 Introduction

Over the past decade, the financial service industry has witnessed a significant advanced technological transformation, particularly in adopting artificial intelligence (AI) and machine learning (ML; Hilb, 2020; Gonaygunta, 2023). These advanced technologies have revolutionized the industry, from enhancing strategic decision-making to strengthening cybersecurity measures (Johri and Kumar, 2023). On the one hand, AI and ML advanced technologies have facilitated and promoted positive trends, such as the digital transformation of business, the development of real-time threat detection, predictive analytics, automated incident response, advanced fraud detection, continuous learning, and phishing, social engineering defense, etc. (Kuzior et al., 2022; AL-Dosari et al., 2024). Prior studies reported that the expressive advancement of AI and ML technologies offers unprecedented opportunities for efficiency, innovation, and competitive advantages, particularly in cybersecurity, while performing financial transactions (Geluvaraj et al., 2019; Narsimha et al., 2022). On the other hand, this shift to advanced technologies of AI and ML has also led to negative consequences, such as the rise of cybercrime facilitated by increased digital literacy and the decreasing cost of the technology needed to commit such crimes. For instance, malicious software can be purchased on the dark web for as little as USD 1, while personal data can be obtained for just USD 3 (Kuzior et al., 2022). This makes it alarming for anyone to become a cybercriminal or gain access to sensitive data at minimal cost. Amid the broader global cybercrime data, the financial service industry has seen a significant surge in attempted cyberattacks, including phishing attacks, malware, distributed denial of service (DDoS) attacks, data breaches, account takeover fraud, mobile banking fraud, and crypto jacking (Ali et al., 2024). These cyber-threats resulted in a financial loss of $110 million (Grantthornton, 2022). The growing impact of cybercrime and cyberfraud is reflected in statistics showing a significant increase in its adverse effects in recent years. In 2023, the financial service industry experienced over 20,000 cyberattacks worldwide, resulting in cumulative losses of USD 2.5 billion and over 12 billion losses over the past two decades (Statista, 2024). Notably, only the Saudi Arabian financial service industry lost USD 110 million in 2022, the highest among the Gulf Cooperation Council (GCC) countries (Grantthornton, 2022). Overall, the financial service industry stands out for its economic losses from cyber incidents and the sheer volume of attacks it endures, accounting for 22.4% of all attacks. Specifically, 70% of these attacks target banks, 16% focus on insurance companies, and 14% affect other financial institutions (IBM Security, 2022). In addition, Gulyás and Kiss (2023) reported that “over 82% of the financial institutions believe that the cybercriminals have become more sophisticated; malware is used in longer and more complex campaigns” (p.86).

However, to encounter cyberattacks in financial institutions, the Saudi Central Bank (SAMA) launched “Project Aber” to utilize AI and ML advanced technologies to create a central bank digital currency that could be used for the settlement of cross-border payment obligations between commercial banks (SAMA, 2020). In addition, SAMA also proposed and implemented a framework following the four main domains, namely: (i) “Cyber Security Leadership and Governance,” (ii) “Cyber Security Risk Management and Compliance,” (iii) “Cyber Security Operations and Technology,” and (iv) “Third Party Cyber Security” aims to control cyberattacks in financial service industry mainly the commercial banks (SAMA, 2023). Despite these initiatives, Saudi Arabia’s commercial banks face numerous challenges in fully integrating AI and ML technologies into their operations. In this regard, Miller (2022) outlined that the banks struggle with adopting these technologies to enhance cybersecurity due to regulatory challenges, a lack of technical expertise, and the need for strategic direction. Gonaygunta (2023) highlighted that integrating AI and ML into the banking sector to deal with complex problems that impose significant strategic directions, where the role of the Board of Directors (BoDs) becomes essential. In the banking sector, the BoDs are accountable for empowering institutions to navigate the complexities of AI and ML adoption ethically and strategically (Vaca et al., 2022).

Therefore, this study aims to examine the critical role of the BoDs in facilitating the adoption of advanced AI and ML technologies in Saudi Arabia’s commercial banking sector. The study seeks to understand how the BoDs can steer the adoption of these advanced technologies to enhance cybersecurity measures, mitigate risks, and ensure regulatory compliance. By focusing on Saudi Arabia, this research addresses a pressing need for guidance on AI and ML adoption in a region highly targeted by cybercriminals and undergoing rapid digital transformation.

However, the relevance of this study is twofold. First, AI and ML are not just tools for operational efficiency; they are increasingly integral to maintaining cybersecurity in the financial service industry. Saudi Arabian banks, highly exposed to cyberattacks, must prioritize these technologies to avoid sophisticated threats. Second, the role of the BoDs is crucial in navigating the complex regulatory and ethical challenges of AI and ML integration. Previous studies have noted the importance of the BoDs in setting strategic directions, fostering innovation, and ensuring that AI and ML are adopted ethically and effectively (Hilb, 2020; Anh, 2021). This study fills this gap in the literature by examining the BoDs’ role in this critical area of AI and ML, particularly in Saudi Arabia’s unique regulatory and business environment.

Theoretically, this research draws on stakeholder theory to examine the role of the BoDs in adopting advanced AI and ML technologies in Saudi Arabian banks. Stakeholder theory emphasizes the importance of aligning corporate governance practices with the interests of all stakeholders, including policymakers and shareholders (Freeman, 1984). In AI and ML adoption, the BoDs must balance innovation with ethical considerations, such as data privacy, algorithmic transparency, and bias mitigation (Vaca et al., 2022). By employing stakeholder theory, this study provides insights into how Saudi Arabian banks can leverage advanced AI and ML technologies to meet stakeholder expectations, enhance customer satisfaction, and reduce the risk of cyberattacks. Given the evolving cybersecurity landscape and the central role of the BoDs in steering technological adoption, this study aims to investigate the following research questions.

RQ1. What is the role of the BoDs in facilitating the adoption of AI and ML in Saudi Arabian banking sector?

RQ2. How can AI and ML effectively protect Saudi Arabian banking sector from cyberattacks?

The abovementioned research questions were addressed through a qualitative study using in-depth interviews with BoDs of Saudi Arabian banks. The research seeks to understand their perspectives on the strategic importance of AI and ML advanced technologies, the challenges faced in their implementation, and the regulatory frameworks that shape their adoption. Overall, this study contributes to the literature on AI and ML in the financial sector by providing empirical insights into the role of the BoDs in their adoption. It offers practical guidance for practitioners and policymakers in the banking industry on navigating the complexities of AI and ML integration. Specifically, it addresses the need for strategic leadership in AI and ML adoption, especially in the context of increasing cybersecurity threats. This study is particularly relevant for Saudi Arabian banks, as it offers actionable insights into how they can harness AI and ML technologies to stay competitive, enhance security, and ensure regulatory compliance.

This paper is structured as follows. Section 2 presents the literature review and theoretical background. Research methods and results are presented in Sections 3 and 4, respectively. Sections 5 and 6 discuss the results, limitations, and future research suggestions.

## 2 Literature review

### 2.1 Artificial intelligence in the banking sector

Cyberattacks have escalated in frequency, severity, and sophistication (Gilad and Tishler, 2023); among these, the banking sector has emerged as a primary targeted industry (Perera et al., 2022). Noticeably, AI has been categorized as a significant tool in the banking sector that supports identifying and avoiding cyberattacks (Narsimha et al., 2022). Banks have increasingly invested in AI-powered services, such as chatbots and financial management tools, to enhance operational efficiency and protection. Furthermore, adopting AI to combat cyber threats has gained traction within the industry (Geluvaraj et al., 2019). Therefore, recent empirical studies on the field are presented in Table 1.

**Table 1:** Relevant empirical studies.

Author (s)	Objectives	Method	Findings
Alraddadi (2023)	The study aimed to develop an abstraction framework to manage and control cybersecurity threats within Saudi banks, based on the National Institute of Standards and Technology (NIST) Cybersecurity Framework and the ISO/IEC 27001 standards.	Mixed method	The study found that Saudi banks generally have robust cybersecurity measures, but there were gaps in their alignment with international standards. The developed framework helped streamline processes, making them more efficient and cohesive.
Englisch et al. (2023)	The study explored how deep learning can be applied to treasury management in banks, improving decision-making and financial forecasting.	Quantitative	The study concluded that deep learning models significantly enhance the accuracy and efficiency of treasury management.
Gilad and Tishler (2023)	The study aimed to examine how the quality, covertness, and intensity of the use of cyber weapons mitigate the risk of advanced cyberattacks.	Quantitative	The study found that high-quality and covert cyber weapons are more effective in mitigating risks from advanced cyberattacks.
Gonaygunta (2023)	The study explored the factors influencing the adoption of machine learning algorithms to detect cyber threats in the banking industry.	Qualitative	The research revealed that critical factors influencing ML adoption in banks include technological infrastructure, data availability, regulatory requirements, and organizational readiness.
Gulyás and Kiss (2023)	The study analyzed the impact of cyberattacks on financial institutions, mainly how they affect operational stability and financial performance.	Quantitative	The study found that cyberattacks significantly negatively affect the financial stability and reputation of institutions.
Johri and Kumar (2023)	The study aimed to explore customer awareness regarding cybersecurity in the Kingdom of Saudi Arabia, particularly in digital banking transformation.	Quantitative	The study found that digital transformation in Saudi banks progresses rapidly, but customer cybersecurity awareness remains limited.
Narsimha et al. (2022)	The study examined the role of artificial intelligence (AI) and machine learning (ML) in defending against financial fraud in the banking sector.	Qualitative	The study concluded that AI and ML are critical for detecting financial fraud and providing faster, more accurate identification of fraudulent activities.
Perera et al. (2022)	This study investigated the factors contributing to reputational damage in organizations following cyberattacks.	Quantitative	The study identified key factors leading to reputational damage, including negative media coverage and poor crisis management.
Vaca et al. (2022)	The study explored the use of deep learning in finance research, particularly in analyzing the profiles of board members in financial institutions.	Qualitative	The study found that deep learning validly analyzes board profiles, offering predictive insights into governance performance.
Cerchiello et al. (2022)	The study aimed to assess bank distress by combining news data with regular financial data, utilizing artificial intelligence (AI) to improve prediction models for bank failures.	Qualitative	The study found that incorporating news data alongside financial data significantly improves the predictive power of models assessing bank distress.
Anh (2021)	The objective was to determine how governance structures influence the implementation and effectiveness of AI systems in banks.	Quantitative	The study found a positive association between strong corporate governance practices and the successful adoption of AI in the banking sector.
Nicholls et al. (2021)	The study provided a comprehensive survey of how deep learning approaches can combat financial cybercrime in the evolving landscape of financial crime.	Quantitative	The study found that deep learning models are highly effective in identifying complex patterns of financial cybercrime, especially when traditional detection methods fail.
Hilb (2020)	The study explored the potential role of artificial intelligence (AI) in shaping the future of corporate governance, focusing on how AI could influence governance practices and decision-making.	Qualitative	The study concluded that AI has the potential to improve governance by providing more data-driven insights for decision-making, enhancing transparency, and automating routine governance tasks.
Geluvaraj et al. (2019)	The study examined the role of AI, machine learning (ML), and deep learning in shaping the future of cybersecurity, particularly in detecting and preventing cyberattacks.	Qualitative	The study highlighted that AI and ML are crucial in proactively defending against cyber threats, with deep learning providing more advanced capabilities in identifying emerging risks.

Nevertheless, leveraging AI in critical infrastructures, the banking industry presents several challenges, i.e., safety, accuracy, trustworthiness, and security systems to adopt AI tools. Consequently, deploying an effective cyber defense system becomes paramount in bolstering customer trust and ensuring the seamless delivery of banking services (Englisch et al., 2023). Nonetheless, various security concerns, challenges, vulnerabilities, and risks continue to emerge, including the deliberate exploitation of AI technology through cyberattacks, which could lead to substantial destruction or even fatalities (Nicholls et al., 2021). Thus, the various processes and services of AI tools in the banking sector are presented in Table 2.

**Table 2:** Process and services of AI in the banking sector.

Artificial intelligence tolls	Impact
Fraud Detection and Prevention	Identifies unusual transactions, prevents unauthorized access, and flags suspicious activities for further investigation.
Customer Service and Support	Handles common customer inquiries, manages accounts, and provides personalized financial advice.
Algorithmic Trading	Credit, market, and operational risks are evaluated to help make informed decisions and regulatory compliance.
Anti-Money Laundering	Monitors large volumes of transactions, identifies suspicious activities, and reports them to relevant authorities.
Risk Management	Enhances trading strategies, improves execution speed, and increases profitability by identifying market opportunities.
Credit Scoring and Loan Approval	Automates the loan approval process, reduces biases in credit scoring, and speeds up decision-making.
Regulatory Compliance and Reporting	Automates reporting monitors transactions for compliance, and flags potential regulatory breaches.
Predictive Analytics	Forecasts market movements, customer needs, and financial outcomes to guide strategic planning and decision-making.
Chatbots and Virtual Assistants	Offers customer service, handles routine inquiries, and assists with transactions such as balance checks and fund transfers.
Sentiment Analysis	Helps in understanding market trends and investor sentiment, influencing trading strategies and investment decisions.

Compiled by author (2024).

However, the role of the board of directors in adopting AI tools in the banking sector is critical for ensuring the successful integration and utilization of these advanced technologies. The board of directors is responsible for setting the strategic direction and providing oversight, including fostering innovations through adopting AI tools (Vaca et al., 2022). They must understand the potential benefits of AI, such as enhanced efficiency, improved customer service, and better risk management assessment, and incorporate these into the bank’s strategic goals. Board governors play a crucial role in resource allocation, ensuring adequate investment in AI technologies and related infrastructure (Cerchiello et al., 2022). The board of directors must also provide the bank with the necessary talent and expertise to implement and manage AI systems effectively. This involves supporting training and development initiatives and attracting skilled data scientists and AI professionals.

### 2.2 Machine learning in the banking sector

ML has emerged as a transformative technology in the banking sector, offering unprecedented opportunities for enhancing various operations and services (Leo et al., 2019; Habib, 2024). ML algorithms, powered by vast amounts of data, enable banks to extract valuable insights, automate processes, and improve decision-making capabilities. However, ML is extensively utilized by financial institutions to improve various operations, enhance decision-making, and provide better customer service (Polireddi, 2024). ML models have the potential to accurately predict credit defaults by analyzing historical data on customer behavior, transaction patterns, and creditworthiness and assess the risk associated with lending to individual customers or businesses (Patel and Trivedi, 2020). This allows banks to make more informed decisions when granting loans and setting interest rates, ultimately reducing the risk of default and improving overall portfolio performance (Kuzior et al., 2022).

Additionally, fraud detection in the banking sector is another critical area that ML substantially impacts (Donepudi, 2017). ML algorithms, however, can analyze vast volumes of transaction data in real-time, identifying patterns and anomalies indicative of fraudulent activity. By continuously learning from new data, ML models can adapt to emerging fraud schemes and enhance the effectiveness of fraud prevention measures (Chen et al., 2020). Therefore, the critical areas of financial institutions where ML could be implemented are presented in Table 3.

**Table 3:** Key areas of financial institutions.

Machine learning	Application
Credit Scoring and Risk Assessment	Automates the loan approval process, reduces biases in credit scoring, and speeds up decision-making.
Anti-Money Laundering (AML) Compliance	Monitors large volumes of transactions, identifies suspicious activities, and reports them to relevant authorities.
Fraud Detection and Prevention	Identifies unusual transactions, prevents unauthorized access, and flags suspicious activities for further investigation.
Market Sentiment Analysis	Helps in understanding market trends and investor sentiment, influencing trading strategies and investment decisions.
Predictive Analytics	Forecasts market movements, customer needs, and financial outcomes to guide strategic planning and decision-making.
Financial Forecasting	Supports investment strategies, budget planning, and risk assessment by providing accurate financial forecasts.
Loan Default Prediction	Helps manage loan portfolios and mitigate risks by identifying high-risk borrowers.
Regulatory Compliance	Automates reporting monitors transactions for compliance, and flags potential regulatory breaches.
Document and Data Processing	Speeds up tasks such as KYC processes, loan applications, and compliance checks by digitizing and analyzing documents.

Compiled by author (2024).

However, chatbots and virtual assistants powered by natural language processing (NLP) and ML algorithms enable banks to provide personalized and interactive customer support around the clock (Patel and Trivedi, 2020). These AI-powered assistants have the potential to handle routine inquiries, process transactions, and even offer financial advice based on individual preferences and behavior, enhancing the overall customer experience (Rabbani et al., 2023).

## 3 Theoretical background

AI and ML have significantly transformed the banking sector by enabling automated processes, enhancing customer experiences, and improving cybersecurity. The adoption of AI and ML offers both positive and negative effects. On the positive side, AI and ML enhance operational efficiency, support risk management, and improve decision-making through predictive analytics (Narsimha et al., 2022). However, the adverse effects include concerns about data privacy, algorithmic bias, and increased vulnerability to sophisticated cyberattacks if AI systems are compromised (Habib, 2024).

AI applications such as chatbots, fraud detection systems, and automated customer support tools have been integrated into banks to improve service quality and security. For instance, banks use ML algorithms to monitor transaction patterns, detect anomalies, and prevent fraudulent activities (Gonaygunta, 2023). However, these technologies also come with risks, including potential misuse of personal data and the challenges of interpreting AI decisions, which may lack transparency (Al-Nasser Mohammed and Muhammed, 2017).

To obtain the objectives of the present study, the stakeholder theory serves as the theoretical framework for understanding the roles of internal stakeholders in AI and ML adoption. Stakeholder theory, introduced by Freeman (1984), emphasizes that organizations should consider the interests of all parties affected by their decisions. For the banking sector, these internal stakeholders include Members of the Board of Directors (BoD), who are responsible for setting strategic goals and ensuring that AI and ML are adopted ethically and effectively. The BoD, as internal stakeholders, are critical in ensuring that AI and ML are implemented in a manner that aligns with regulatory standards, protects customers, and promotes transparency (Güngör, 2020). The focus on the BoD’s role in AI and ML adoption explores their strategic decision-making in enhancing cybersecurity while managing risks such as data breaches and algorithmic biases (Vaca et al., 2022).

## 4 Risks and criticisms of AI and ML in banking

AI and ML applications in banking present risks such as algorithmic errors, cyber threats, and ethical concerns. Algorithmic decision-making may introduce biases that negatively affect customers, particularly in loan approvals and fraud detection areas. Furthermore, AI-driven systems can become targets for cybercriminals, who may exploit vulnerabilities in the algorithms or data infrastructure (Güngör, 2020). The financial sector’s transition toward AI and ML adoption in Saudi Arabia is complicated by regulatory challenges, limited technological expertise, and the need for robust governance frameworks. Stakeholder theory provides a suitable lens to assess how the BoD, as key internal stakeholders, manages these risks while navigating the technological transformation (Laine et al., 2024). Therefore, the present study focuses on AI and ML applications such as fraud detection systems, automated customer support, and risk management tools. These applications illustrate the growing role of AI and ML in transforming banking operations, but they also require careful management to ensure security and trust in financial systems.

Noticeably, the concepts of stakeholder theory are increasingly applied in countries like Germany, Sweden, and Japan, where boards of directors have a significant role in organizational operations. This approach empowers all stakeholders to participate in the organization’s current and future activities. In conclusion, stakeholder theory provides a suitable framework for understanding the adoption of AI and ML practices in the context of this study.

## 5 Method

This study adopts an exploratory qualitative research design to examine the role of the BoD in AI and ML adoption within Saudi Arabian banks. Using qualitative methods, such as in-depth interviews, allows for a detailed examination of the perceptions, strategies, and challenges BoD members face in adopting AI and ML for cybersecurity. One cybersecurity management professional and three professional academic researchers have prepared and validated the interview protocols. However, the experts recommended minor modifications to the interview protocol, and we modified the items accordingly. Overall, through the above process, we ensure the validity and reliability of the interview questions.

### 5.1 Data collection

Participants were recruited through email and face-to-face communication, followed by a flexible scheduling process to accommodate their availability. Interview durations ranged from 45 min to 1 h, and the interviews were conducted through Google Meet for remote participants or in person. Interviews were recorded with the participant’s consent, ensuring accurate transcription and analysis. Therefore, the interview protocol focused on three key areas such as (i) the strategic importance of AI and ML in banking, (ii) the BoD’s role in overseeing AI and ML adoption, and (ii) challenges in implementing AI and ML to enhance cybersecurity. However, participants were encouraged to interrupt the interviewer for clarification and were assured that there were no right or wrong answers. The interviewer fostered a relaxed, informal atmosphere to promote open and honest discussion. Participants were informed that their responses would be treated with confidentiality and stored securely on a password-protected computer. To maintain anonymity, participant information was identified by a number rather than their name.

### 5.2 Data analysis

In this study, we utilize thematic analysis, grounded in a realist ontology, which assumes that language reflects reality and focuses on participants’ thoughts and statements. The approach to coding and theme development follows a flexible method, as Mahmood et al. (2023) suggested. According to AL-Dosari et al. (2024), interview questions are often recommended to guide the development of themes, a technique also applied in this study. The interview questions were designed based on existing theories and relevant literature, expecting that they would partly inform the significant themes. However, the coding and final theme development were conducted independently of the interview structure to minimize subjectivity bias and ensure a more objective analysis.

The codes were developed inductively, meaning the data served as the analysis’s starting point. Semantic coding was used to capture explicit meanings in the data, limiting the influence of researcher subjectivity. The study was carried out in several stages, as outlined by Terry et al. (2017): familiarizing with the data, generating codes, constructing themes, reviewing potential themes, and defining final themes. The data organization was facilitated by NVIVO 12 software, which helped structure the codes. The study also followed the six-step qualitative analysis framework by Clarke and Braun (2013), provided below.

(i) Familiarization with the data: Immersing oneself in the dataset through comprehensive review and multiple readings.
(ii) Coding: Creating concise labels (coding) to identify pertinent data elements crucial for addressing research questions.
(iii) Generating initial themes: Using coded data to develop preliminary themes, then gathering relevant information for each potential theme to assess its viability.
(iv) Reviewing themes: Evaluating prospective themes against the dataset, refining or discarding them based on their alignment with the data and research objectives.
(v) Defining and naming themes: Scrutinizing each theme to delineate its scope, purpose, and narrative, accompanied by assigning descriptive labels to subtopics.
(vi) Writing up: Integrating analytical narratives with data excerpts and contextualizing findings within existing literature during the interpretation phase.

### 5.3 Demographic characteristic

To obtain the objectives of the present study, 17 semi-structured interviews were conducted with the BoDs from retail banks in Saudi Arabia. Demographic information of the respondents, including gender, age, position, nationality, and region, was identified. Since the personal information of the interviewees was confidential, the names were coded from “BoM1” to “BoM17.” “BoM” stands for Board of Directors, and numbers refer to the order in which interviews were conducted. Therefore, the demographic characteristics of the respondents are presented in Table 4.

**Table 4:** Demographic characteristics of the board of members.

No	Code	Position	Gender	Qualification	Nationality	Classification	Region	Age
1	BoD1	Board of Directors	Male	Postgraduate	Saudi	Non-Executive	East	45
2	BoD 2	Board of Directors	Male	Postgraduate	Saudi	Independent	West	44
3	BoD 3	Board of Directors	Male	Postgraduate	Saudi	Independent	Middle	40
4	BoD 4	Board of Directors	Male	Postgraduate	Saudi	Independent	East	45
5	BoD 5	Board of Directors	Male	Undergraduate	Saudi	Independent	Middle	51
6	BoD 6	Board of Directors	Male	Undergraduate	Saudi	Independent	East	43
7	BoD 7	Board of Directors	Male	Postgraduate	Saudi	Non-Executive	Middle	52
8	BoD 8	Board of Directors	Male	Postgraduate	Saudi	Independent	East	62
9	BoD 9	Board of Directors	Male	Undergraduate	Saudi	Independent	West	50
10	BoD 10	Board of Directors	Male	Undergraduate	Saudi	Independent	Middle	59
11	BoD 11	Board of Directors	Male	Postgraduate	Saudi	Independent	West	64
12	BoD 12	Board of Directors	Female	Postgraduate	Saudi	Non-Executive	West	29
13	BoD 13	Board of Directors	Male	Postgraduate	Saudi	Executive	West	57
14	BoD 14	Board of Directors	Male	Undergraduate	Saudi	Non-Executive	Middle	32
15	BoD 15	Board of Directors	Female	Postgraduate	Saudi	Executive	West	63
16	BoD 16	Board of Directors	Male	Postgraduate	Saudi	Non-Executive	West	50
17	BoD 17	Board of Directors	Male	Undergraduate	Saudi	Executive	West	51

Compiled by author (2024).

## 6 Results

This section presents the findings from the qualitative interviews with 17 BoDs in the banking sector of Saudi Arabia. The interviews focused on the role of BoDs in facilitating the adoption of AI and ML in the Saudi Arabian banking sector to strengthen cybersecurity and prevent cyberattacks. The findings are organized around two key research questions (RQ1 and RQ2), with insights into how AI and ML are implemented to address cybersecurity and fraud detection challenges.

Findings on RQ1: RQ1. What is the role of the BoDs in facilitating the adoption of AI and ML in Saudi Arabian banking sector?

In exploring the first theme of the present study, interviewees highlighted the BoDs’ strategic role in driving the adoption of AI and ML within Saudi Arabian banks. The BoDs’ influence was crucial in setting the vision for digital transformation, allocating resources, and addressing these advanced technologies’ regulatory and ethical complexities. This theme centers on the BoDs’ capacity to champion AI and ML adoption while balancing innovation with regulatory compliance and moral integrity. However, several interviewees emphasized that a clear vision from the BoDs is essential for successfully adopting AI and ML in Saudi banks. One board member pointed out that the BoDs’ primary responsibility is to set a strategic agenda that aligns with Saudi Arabia’s Vision 2030 goals, pushing for digitalization and technological advancement across the banking sector:

*“Our role is to ensure that our bank’s strategic goals align with Vision 2030. AI and ML are at the forefront of these goals, and the board’s responsible for leading this transformation with clear directives and well-defined objectives (BoD1).”*

Another interviewee echoed the importance of the BoDs in establishing a strategic roadmap for AI and ML, emphasizing that without BoDs leadership, the integration of these technologies would likely stall:

*“If the board is not committed to an AI-driven strategy, progress halts. We provide the direction, and our commitment trickles down through the organization, encouraging buy-in from all levels (BoD2).”*

Therefore, interviewees also discussed the BoDs’ role in securing and allocating resources for AI and ML projects, which often require significant financial investments. Many Saudi banks face budgetary constraints, and prioritizing resources for AI and ML can be challenging. One board member highlighted the BoDs’ responsibility to secure adequate funding:

*“AI and ML adoption requires significant upfront investment, not just in technology but also in upskilling our workforce and strengthening infrastructure. It’s the board’s job to ensure these resources are available (BoD7).”*

Another respondent (BoD8) noted that the BoDs must be proactive in fostering partnerships with AI technology providers, which can help offset some of the resource limitations and provide access to cutting-edge tools and expertise:

*“Collaborating with tech providers allows us to leverage their expertise and access the latest AI solutions without exhausting our resources. These partnerships are something the board actively pursues (BoD8).”*

Additionally, the interviewees highlighted that AI and ML implementation in Saudi banks has significant regulatory and ethical challenges. The BoDs’ role in navigating these issues was widely recognized as critical. With stringent regulations, particularly around data privacy and anti-money laundering (AML) requirements, BoDs noted the importance of setting up robust governance structures. One interviewee stressed this regulatory aspect:

*“In a highly regulated industry like banking, the board has to ensure that any AI or ML initiative complies with legal requirements. We work closely with legal and compliance teams to ensure our tech use does not expose us to regulatory risks (BoD11).”*

The interviewees also mentioned the ethical implications of AI, including concerns about bias and transparency. Board members see themselves as gatekeepers who must oversee AI implementations that uphold fairness and avoid discrimination:

*“We cannot afford to have biased algorithms or opaque decision-making processes. As board members, we are responsible for ensuring our AI systems are transparent and fair, especially given the sensitive nature of financial data (BoD13).”*

The BoDs’ role in fostering an organizational culture receptive to AI and ML was also emphasized. Many interviewees noted that adopting AI and ML requires a shift in mindset across all levels of the organization, particularly given the conservative nature of the Saudi banking sector. One respondent mentioned the importance of change management and cultural adaptation led by the BoD:

*“AI adoption is not just about technology; it’s a cultural shift. The board must champion this change and create an environment encouraging innovation and agility (BoD13).”*

Another board member shared insights into how the BoDs’ support for training and upskilling initiatives is critical for long-term success:

*“Our workforce needs to be ready for the AI era. The board actively promotes training and development programs to help employees adapt to these technologies, which is essential for sustainable adoption (BoD15).”*

However, some interviewees reflected on the differences between the BoDs’ role in Saudi Arabia and other countries, noting that Saudi boards may face unique cultural and regulatory challenges. In contrast, banks in countries like the United States and Germany may experience fewer regulatory hurdles and a more flexible environment for AI and ML adoption. One board member commented:

*“In places like the U.S., boards have more room to experiment and innovate with AI. The conservative banking culture and regulatory caution in Saudi Arabia can slow things down. But Vision 2030 is helping us move in the right direction (BoD16).”*

Findings on RQ2: RQ2. How can AI and ML effectively protect Saudi Arabian banking sector from cyberattacks?

The interviewees consistently emphasized that AI-based solutions are widely adopted in Saudi Arabian banks to combat cyber threats, mainly distributed denial of service (DDoS) attacks. These solutions leverage deep learning and artificial neural networks, providing greater flexibility and robustness than conventional systems. AI’s role in cybersecurity within Saudi Arabian banks is crucial, primarily due to its ability to automate security operations and streamline processes like network traffic monitoring, anomaly detection, and malicious activity identification. One participant noted:

*“The ability of AI to scale our defenses against DDoS attacks is unparalleled. We’ve integrated a genetic algorithm for traffic analysis, which allows us to adjust to fluctuating demands and threats much faster than before (BoD3).”*

Another interviewee discussed how AI-based Optical Character Recognition (OCR) systems enhance fraud detection and compliance efforts:

*“We’ve employed AI in our KYC procedures. It has revolutionized how we handle document scanning and verification, reducing manual errors and speeding up compliance tasks (BoD3).”*

AI technologies have enabled banks to meet regulatory frameworks, such as SAMA’s Counter-Fraud guidelines, which require comprehensive governance, detection, and prevention mechanisms. A significant advantage noted by interviewees is AI’s ability to mine large datasets in real-time, facilitating swift responses to threats:

*“AI’s ability to handle enormous volumes of transactional data allows us to stay ahead of cybercriminals, who constantly evolve their methods (BoD4).”*

Furthermore, Machine learning (ML) has also emerged as a critical tool in cybersecurity. The interviewees reported that ML-based algorithms, such as deep learning and neural networks, are highly effective in detecting complex threats like DDoS attacks. ML models enable banks to analyze vast amounts of log data, providing real-time insights and enhancing fraud prevention capabilities beyond traditional systems. One interviewee stated:

*“With ML, we can identify patterns that would be impossible to detect manually. This capability is invaluable in our fight against fraud, especially in credit card transactions (BoD5).”*

However, interviewees also highlighted challenges in implementing ML-powered systems. A recurring issue was the absence of a well-defined AI strategy and a lack of comprehensive technological infrastructure within banks, leading to inefficiencies in fully utilizing ML’s potential:

*“The biggest obstacle is our outdated tech foundation. Without a modern infrastructure, we cannot fully capitalize on ML’s capabilities (BoD6).”*

Ethical concerns regarding ML were also discussed, particularly around risks of data manipulation and unintended biases. One interviewee mentioned the dangers posed by ethical hacking and social engineering:

*“Our biggest fear is that ML systems can be exploited by cybercriminals, who could manipulate datasets to launch more realistic and damaging attacks. We’ve had instances where ethical hackers were able to bypass our systems (BoD9).”*

Despite these challenges, the interviewees underscored ML’s strength in providing real-time predictive analytics, especially in fraud detection:

*“The speed at which ML analyzes data is key. It allows us to predict potential threats and respond before they cause serious damage. This capability is transformative in credit card fraud detection (BoD11).”*

In comparing AI and ML adoption in Saudi Arabian banks to international contexts, interviewees highlighted several cultural, political, and regulatory differences. One interviewee noted that the relatively conservative banking culture in Saudi Arabia, combined with stringent regulatory controls, has slowed AI and ML integration compared to more liberal markets in Western Europe or North America:

*“We’re cautiously optimistic, but our pace of AI and ML adoption is hindered by cultural factors and regulatory caution, especially compared to the United States or Europe (BoD15).”*

However, Saudi Arabia’s Vision 2030 initiative is viewed as a turning point. With the government’s focus on digital transformation, Saudi Arabian banks expect that the pace of AI and ML integration will accelerate, supported by additional resources and infrastructure:

*“Vision 2030 is encouraging us to advance our digital capabilities. We anticipate that with governmental support, we can bridge the infrastructure gap and accelerate AI and ML adoption to meet international standards (BoD13).”*

These insights underscore the influence of external factors on AI and ML adoption within Saudi Arabian banks, highlighting the challenges and opportunities associated with the country’s digital transformation efforts.

## 7 Discussion

The findings of this study underscore the critical role that artificial intelligence (AI) and machine learning (ML) play in enhancing cybersecurity within Saudi Arabian banks. By investigating the perceptions of the board of directors (BoD), this study highlights both the opportunities and challenges associated with AI and ML adoption. The results suggest that AI and ML are indispensable tools for modern banking, offering advanced threat detection, fraud prevention, and compliance with regulatory standards.

However, the study also points to significant barriers, such as the absence of a cohesive AI strategy, limitations in technological infrastructure, and ethical concerns. Several Saudi Arabian banks lack a comprehensive AI strategy, which limits the full realization of these technologies’ potential (Perera et al., 2022). The BoD’s involvement in creating a clear, unified strategy for AI adoption is essential to overcome this barrier. In this view, Tariq et al. (2021) highlighted that the outdated infrastructure and insufficient data foundations hinder banks’ ability to utilize advanced AI and ML technologies fully. The findings indicate a need for significant investments in technological infrastructure, led by strategic guidance from the BoDs, to support advanced AI and ML initiatives. Furthermore, AI and ML systems carry algorithmic bias risks and cyber exploitation vulnerability, necessitating stronger ethical oversight and legal frameworks (Gandhi et al., 2024). The present study identified that the BoDs must ensure that AI systems are transparent, fair, and compliant with regulations, as ethical governance is crucial for sustaining public trust in AI-driven banking solutions.

### 7.1 Practical implications

The practical implications of this study are substantial for both banks and regulatory bodies like the Saudi Arabian Monetary Authority (SAMA). AI and ML have emerged as vital technologies for enhancing cybersecurity, with significant fraud detection and compliance applications. AI solutions like IBM AI and deep-learning OCR tools can streamline compliance with tasks like Know Your Customer (KYC) verification and fraud detection, providing scalable solutions that align with regulatory demands (Polireddi, 2024). These insights suggest that banks need to integrate advanced AI and ML tools to remain competitive in a rapidly evolving financial landscape. The findings reinforce the need for Saudi Arabian banks to partner with third-party AI providers to access cutting-edge technologies and mitigate internal limitations. Additionally, upskilling the workforce is essential for effectively managing these complex AI systems (Rabbani et al., 2023). The BoDs’ role in supporting workforce development initiatives is crucial to ensure a well-prepared workforce that can adapt to the technological advancements associated with AI and ML adoption. For regulatory authorities like SAMA, this study highlights the importance of developing a framework that guides ethical AI use in the banking sector. SAMA’s regulatory oversight ensures that AI adoption aligns with anti-money laundering (AML) and fraud detection standards, fostering a more secure banking environment (Gandhi et al., 2024).

### 7.2 Managerial implications

The results of this study offer several managerial insights for banking executives, especially the BoDs, in overseeing AI and ML integration. The BoDs’ proactive role in promoting digital transformation and aligning AI strategies with broader organizational goals is essential. This study also outlined the importance of strategically diverse BoDs, which enhances decision-making in critical areas such as cybersecurity and regulatory compliance (Vaca et al., 2022). Such diversity is beneficial for strategic planning and aligns with the goals of Saudi Vision 2030, which promotes digital innovation within the financial sector (Alraddadi, 2023). Additionally, the BoDs must address ethical considerations in AI applications, including privacy concerns, algorithmic bias, and transparency. Managers must establish robust ethical frameworks to prevent data misuse and ensure fair and transparent decision-making processes (Rodrigues et al., 2022). The BoDs’ influence in promoting a culture of ethical AI adoption can also guide organizations toward sustainable, responsible innovation.

### 7.3 Theoretical implications

From a theoretical perspective, this study extends stakeholder theory by illustrating the influence of internal stakeholders (BoDs) on AI and ML adoption in the banking sector. Consistent with Freeman’s (1984) stakeholder theory, the findings emphasize the need to address the interests and responsibilities of all parties impacted by technological advancements (Güngör, 2020; Rodrigues et al., 2022). In AI and ML, adoption goes beyond operational efficiency; it requires a balance of ethical and regulatory considerations impacting customers and regulatory authorities.

The BoDs’ role in setting a strategic vision for AI and ML adoption aligns with previous research that stresses the importance of governance structures in facilitating technology integration (Vaca et al., 2022). The findings suggest that diverse BoDs can provide broader perspectives on risk management and innovation, which enhances decision-making for AI-related initiatives. Furthermore, this study highlights the ethical risks associated with AI, such as data privacy and algorithmic bias, extending the discussion on the need for robust governance frameworks to mitigate these risks (Perera et al., 2022).

### 7.4 Limitations and future research agenda

This study is not without limitations. The present study is instructive concerning the role of AI and ML in enhancing cybersecurity within the banking sector in Saudi Arabia, but there are a few limitations. First, there might be biases in the responses of expert interviews and, therefore, in the qualitative data, sample sizes of interviewees may limit the generalization of findings within banking in Saudi Arabia. Thus, the other critical dimensions of technological innovation and risk management within the banking sector may be shadowed if the focus is strictly on AI and ML applications in banks’ cybersecurity systems. These could be significantly better addressed in future research endeavors if a mixed-method approach is adopted that taps into qualitative insights from expert interviews, therefore informing a quantitative data analysis of cybersecurity metrics and performance indicators. An effective mixed method, hence, may combine qualitative insights from expert interviews with quantitative data on cybersecurity metrics and performance indicators on AI and ML technology by deepening our understanding of how effective they can be in minimizing cyber threats and improving overall cybersecurity postures in the banking industry of Saudi Arabia. Moreover, it will also be interesting to find out whether potential future research further expounds on how banks’ implementation of AI and ML solutions in cybersecurity poses challenges and barriers to issues that emanate from data privacy, regulatory compliance, and resources. Realization of the difficulties, therefore, would allow banks to use AI technologies to maximize the return on improved cybersecurity outcomes. Consequently, it would be necessary to have long-term case histories to document the ever-evolving nature of applications of AI and ML in the cybersecurity domain and appraise their long-term effectiveness in quelling emerging security threats. Within this line, longitudinal studies could also investigate the scalability and sustainability of AI-powered solutions in cybersecurity, including their attendant implications for the resilience of the Saudi Arabian banking sector against evolving cyber risks. Finally, research should also identify possible synergies that can exist between AI, ML, and other new technologies, such as blockchain and quantum computing, in the pursuit of strengthened cybersecurity defenses and enabled new, innovative risk management strategies. With this all-inclusive approach to technological innovation and risk mitigation, the Saudi Arabian banking sector is bound to be the new vanguard in cybersecurity resilience, protecting continued trust and confidence from its customers and stakeholders within the digital era.

## 8 Conclusion

AI-powered solutions have enabled banks to streamline KYC procedures and improve internal fraud detection mechanisms, bolstering overall risk management capabilities. However, despite the significant benefits offered by AI and ML technologies, banks in Saudi Arabia face several challenges in their implementation and utilization. These challenges include the lack of a well-defined AI and ML strategy, inadequate technology infrastructure, and concerns related to data privacy and ethical considerations. Moreover, the reliance on third-party AI and ML solutions and the shortage of skilled cybersecurity professionals poses additional obstacles to effective cybersecurity management. Addressing these challenges will be crucial for the Saudi Arabian banking sector to harness the full potential of AI and ML in bolstering cybersecurity defenses. This will require concerted efforts from industry stakeholders and policymakers to develop robust AI and ML governance frameworks, invest in cybersecurity education and training programs, and foster collaboration between banks and technology providers. Moreover, AI and ML-powered security solutions can autonomously adapt and evolve in response to cyber threats, continuously learning from past incidents and refining their detection capabilities. By leveraging ML models to analyze historical attack data and identify patterns, financial institutions can enhance their ability to detect and mitigate known and unknown cyber threats, bolstering their overall resilience against cyber-attacks. While AI and ML promise to strengthen cybersecurity in the Saudi Arabian banking sector, their successful implementation hinges on overcoming various organizational, technical, and regulatory challenges. By embracing a proactive and collaborative approach to cybersecurity management, Saudi Arabian banks can strengthen their resilience against cyber threats and safeguard the integrity and trust of the financial system.

## Data Availability

The raw data supporting the conclusions of this article will be made available by the authors, without undue reservation.
